# N-3 Fatty Acid Supplementation Impacts Protein Metabolism Faster Than it Lowers Proinflammatory Cytokines in Advanced Breast Cancer Patients: Natural ^15^N/^14^N Variations during a Clinical Trial

**DOI:** 10.3390/metabo12100899

**Published:** 2022-09-24

**Authors:** Olivier L. Mantha, Régis Hankard, Illa Tea, Anne-Marie Schiphorst, Jean-François Dumas, Virginie Berger, Caroline Goupille, Philippe Bougnoux, Arnaud De Luca

**Affiliations:** 1 Nutrition, Growth and Cancer (N2C) UMR 1069, University of Tours, INSERM, 37032 Tours, France; 2 Nantes University, CNRS, CEISAM, UMR6230, F-44000 Nantes, France; 3 Department of Patient Education, Institut de Cancérologie de l’Ouest, 49055 Angers, France; 4 Department of Gynecology, Centre Hospitalier Régional Universitaire de Tours, Hôpital Bretonneau, 2 Boulevard Tonnellé, 37044 Tours, France

**Keywords:** nitrogen-15 isotopic abundance, carbon-13 isotopic abundance, n-3 fatty acids, EPA, DHA, protein and amino acid metabolism, inflammation, breast cancer

## Abstract

While clinical evidence remains limited, an extensive amount of research suggests a beneficial role of n-3 polyunsaturated fatty acid supplementation in cancer treatment. One potential benefit is an improvement of protein homeostasis, but how protein metabolism depends on proinflammatory cytokines in this context remains unclear. Here, using the natural abundance of the stable isotopes of nitrogen as a marker of changes in protein metabolism during a randomized, double-blind, controlled clinical trial, we show that protein homeostasis is affected way faster than proinflammatory cytokines in metastatic breast cancer patients supplemented with n-3 polyunsaturated fatty acids. We provide some evidence that this response is unrelated to major changes in whole-body substrate oxidation. In addition, we demonstrate that more fatty acids were impacted by metabolic regulations than by differences in their intake levels during the supplementation. This study documents that the percentage of patients that complied with the supplementation decreased with time, making compliance assessment crucial for the kinetic analysis of the metabolic and inflammatory responses. Our results highlight the time-dependent nature of metabolic and inflammatory changes during long-chain n-3 fatty acid supplementation.

## 1. Introduction

A growing body of preclinical evidence suggests that the therapeutic use of n-3 polyunsaturated fatty acid(s) (PUFA) could improve a variety of clinical outcomes in cancer patients. Yet, clinical evidence remains limited [1]. There are hypotheses and identified mechanisms for both effects of n-3 PUFA at the tumor and whole-body levels. Interindividual variability in the response to n-3 PUFA intake has been documented [2] and can be attributed, at least partly, to polymorphisms affecting their metabolism [3,4,5] and to compliance. Thus, it is becoming increasingly clear that reinforcing the body of clinical evidence will require good knowledge about compliance and individual response to n-3 PUFA.

In this context, the effects of n-3 PUFA include changes in protein metabolism that could alleviate lean mass loss during advanced cancer [6]. How these changes in protein metabolism relate to inflammation remains unclear. Whereas supplementation with n-3 PUFA can lower inflammation [7], and proinflammatory cytokines affect protein metabolism [8], there have been indications of improved muscle protein homeostasis without concomitant modulation of inflammation after n-3 PUFA supplementation, suggesting an anabolic effect unrelated to systemic inflammation [6,9,10,11,12,13]. Simple markers of such a metabolic response would benefit future clinical and preclinical research efforts. Here, we investigated the potential of natural isotopic variations.

Using a randomized, double-blind, controlled trial, we investigated how n-3 PUFA supplementation differentially impacts proinflammatory cytokines and protein metabolism. A supplement containing fish oil, rich in the long-chain n-3 PUFA eicosapentaenoic acid (20:5n-3) (EPA) and docosahexaenoic acid (22:6n-3) (DHA), was tested against a control supplement matching caloric and macronutrient intake. Interindividual variability in compliance and supplementation dose-response was assessed. While protein metabolism is typically investigated with the administration of an isotopically enriched tracer [9], the natural abundance of stable isotopes of nitrogen, more viewed as a marker of dietary exposure to isotopically distinct food sources [14,15,16], also varies with protein metabolism. Relative to dietary input, the heavy stable isotope of nitrogen (^15^N) bioaccumulates compared to its lighter counterpart (^14^N) to an extent that has been shown to depend on protein and amino acid metabolism [17,18,19], due to isotopic fractionation during certain metabolic reactions [20,21]. Therefore, we measured natural ^15^N/^14^N variations that turned out to rapidly occurred after supplementation onset, establishing that changes in protein metabolism are unrelated to proinflammatory cytokines that were only later reduced. We also measured natural variations in the abundance of stable isotopes of carbon (^13^C/^12^C), yielding evidence that changes in protein metabolism during long-chain n-3 PUFA supplementation are unrelated to major changes in whole-body substrate oxidation. Altogether, our results further show that natural isotopic abundances (expressed as δ^15^N and δ^13^C) are useful markers in clinical research efforts. Finally, we identified which specific fatty acid(s) (FA) are involved in the metabolic response observed and explored the role of desaturase enzymes.

## 2. Material and Methods

### 2.1. Study Design and Participants

This study was a randomized, double-blind, controlled, phase III clinical trial. Eligible patients were women ˃18 years of age with a histologically confirmed metastatic breast cancer (HER2-negative and HR-positive); ≥1 measurable lesion according to RECIST 1.1 criteria; planned first-line chemotherapy; a life expectancy of ˃3 months; a WHO performance status ≤2; and acceptable hematologic, liver hepatologic, and renal parameters. Exclusion criteria were triple-negative or HER2 positive breast cancers; non-measurable disease; symptomatic central nervous system metastasis; a WHO performance status ˃2; previous chemotherapy for metastatic breast cancer; a body mass index ˃35; the presence of another invasive cancer; intolerance to milk protein; a clinically significant or uncontrolled cardiac disease or uncontrolled hypertension; and childbearing potential without efficient contraceptive use, pregnancy, and breastfeeding. Signed informed consent was obtained and the study was approved by an ethics committee (CPP Ouest II-Angers). Enrolled patients were randomly assigned to either the control group or the fish oil supplementation group using a web-based service. Patients were stratified by site, by the presence of ˃1 metastatic site or hepatic metastases, and by a chemoresistance index based on the occurrence of metastasis/relapse <6 months after the end of locoregional adjuvant treatment. The primary outcome of this trial was progression-free survival at 4 months with 210 patients initially scheduled for inclusion. However, the start-up that was producing the fish oil and control supplements had to cease its activity resulting in the early termination of the trial. The sample size was too small to adequately analyze survival, especially after accounting for compliance (63 patients included). This study was registered at clinicaltrials.gov as NCT01548534.

**Figure 1:**
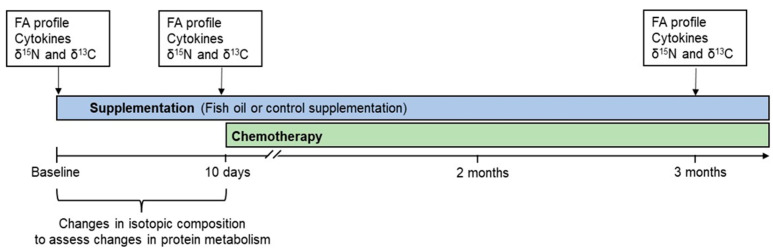
Study timeline. FA, fatty acid.

**Figure 2:**
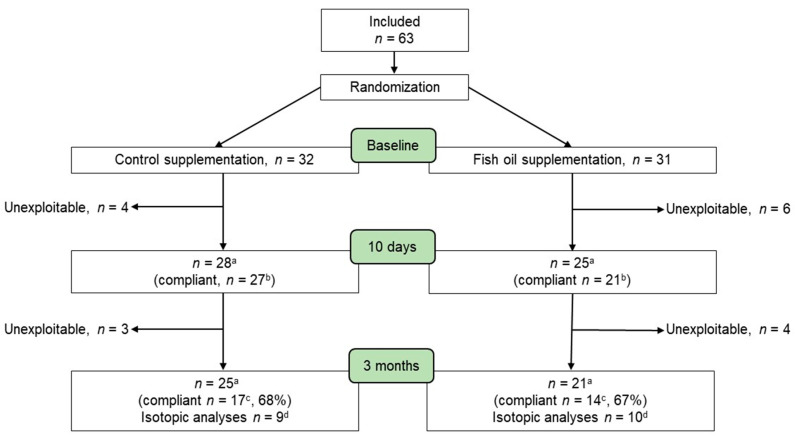
Study participant flow chart. Unexploitable patients either withdrew from the trial, were excluded due to an early interruption of chemotherapy, or had a missing sample. ^a^ Sample used for the analyses of the changes in plasma fatty acids presented in Figure 3. ^b^ Sample of compliant patients used for the partial least squares discriminant analysis. ^c^ Sample of compliant patients used for the kinetic analyses of plasma fatty acids and cytokines. ^d^ Compliant patient subsample used for the isotopic analyses.

Plasma FA profile, proinflammatory cytokine levels, and natural isotopic abundances were determined at randomization before nutritional supplementation was initiated (baseline) and 10 days later just before chemotherapy onset and after 3 months (Figure 1), using blood (5 mL) collected in lithium heparin tubes. Changes in protein metabolism were assessed using natural δ^15^N variations. Changes in isotopic compositions occurring from supplementation onset to chemotherapy initiation (Figure 1) were used to normalize for differences in dietary input and isolate the effect of metabolism. Natural δ^13^C variations were also determined, allowing us to gain further insights into concomitant changes in whole-body metabolism. A total of 63 patients were included in the study and allocated to the control (*n* = 32) and fish oil (*n* = 31) groups (Figure 2). From supplementation onset to 3 months later, 7 and 10 patients in the control and fish oil groups, respectively, either withdrew from the trial, were excluded due to an early interruption of chemotherapy, or had a missing sample. Forty-six patients consequently completed the study (Figure 2).

### 2.2. Supplements

Each patient was instructed to consume 3 cans per day of a supplement (Castase, Nutryalis Medical Nutrition, St-Grégoire, France) that contained fish oil or a control supplement. Patients were instructed to start each meal with a supplement drink, to complete their meal with their usual diet, and avoid changing their food choices. Both the supplement containing fish oil and the control supplement had the same macronutrient composition and caloric contents (Table 1). Copra oil was used as an alternative to fish oil in the control supplement to match lipid content without providing more n-6 PUFA and less of the nutritionally essential 18:3n-3, thus minimizing the impact on n-3 PUFA levels. Detailed FA compositions of the supplements are described in Table S1. The fish oil supplement, while having the same total lipid content as the control supplement, was richer in PUFA and monounsaturated FA (MUFA) and contained less saturated FA. The control supplement was richer in medium-chain fatty acids. δ^15^N was the same in both the fish oil and control supplements because they contained the same proteins and amino acids. δ^13^C was lower by 0.8‰ in the control supplement due to its different FA profile. Upon randomization, patients were provided with 5 days of supplements and more supplements were then delivered to the patients by the manufacturer. Compliance was assessed using patient diaries and FA incorporation into plasma phospholipids (PL) as explained in Section 3.1.

### 2.3. Fatty Acid Profile

Plasma was separated by centrifugation. Plasma lipids were extracted using an adapted version of commonly employed protocols [22]. Briefly, lipids were extracted from 0.5 mL of plasma with 3.7 mL of chloroform and 1.5 mL of methanol. The mixture was centrifugated (2200 g, 10 min) and the bottom organic layer was collected and filtered through glass wool and dried with sodium sulfate. A total of 3 mL of chloroform/methanol (2/1, *v*/*v*) and 0.5 mL of H_2_O were added to the aqueous phase. The mixture was centrifugated (2200 g, 10 min) and the organic phases from both extractions were pooled and concentrated using centrifugal evaporation. Plasma PL were purified from extracted lipids using a one-dimensional thin-layer chromatography. Briefly, the lipid extract (40 μL) was applied to a silica-coated plate that was developed in hexane:ether:acetic acid (70/30/1)(*v/v*/*v*). After migration, lipid species were revealed using 2′,7′-dichlorofluorescein and viewed under ultra-violet light. Extracted plasma PL were then transmethylated using 14% boron trifluoride in methanol (90 min, 100 °C). After extraction with hexane, FA methyl esters were analyzed by gas chromatography–flame ionization detection (GC-2010 Plus, Schimadzu, France) (BPX70 column, 60 m × id 0.25 mm, SGE, Courtaboeuf, France) as previously described [23]. For supplement analyses, triglycerides were separated by thin layer chromatography and the concentration of transmethylated FA was avoided to allow the analysis of the more volatile short-chain FA (Table S1). FA chromatographic peaks were identified using retention times of standards and expressed as a percentage of the total chromatographic area using GCsolution software (Shimadzu, France):

FA proportion (area%) = 100 × AUC_FA_/AUC_total_, (1)

where AUC_FA_ is the area under the curve of the chromatographic peak of an FA and AUC_total_ is the sum of the areas of all the chromatographic peaks during a predetermined time window. The identified AUC_FA_ accounted for more than 95% of AUC_total_. Typical chromatograms are presented in Figure S1. Sample concentrations were adjusted to obtain a similar AUC_total_ and a quality control process was used: the integration window and identification of each AUC_FA_ were verified and adjusted by the same person (C.G.). Indices of enzymatic activity were obtained from ratios between the area% of precursors and products (18:1n-9/18:0 for the Δ9-desaturase (Stearoyl-CoA desaturase 1, SCD1); 18:3n-6/18:2n-6 for the Δ6-desaturase (fatty acid desaturase 2, FADS2); and 20:4n-6/20:3n-6 for the Δ5-desaturase (fatty acid desaturase 1, FADS1)).

### 2.4. Proinflammatory Cytokines

Plasma interleukin-6 (IL-6) and tumor necrosis factor-alpha (TNF-α) were measured by ELISA (R&D Systems, Abingdon, United Kingdom).

### 2.5. Isotopic Analyses

For a subset of compliant patients (Figure 2), plasma was lyophilized and analyzed using elemental analyzer isotope ratio mass spectrometry (EA-IRMS). From each sample, ~0.7 mg was weighed with a 10^−5^ g precision balance (Ohaus Discovery DV215CD, Pine Brook, NJ, USA) into each of three tin capsules (solids “light” 5 × 9 mm, Thermo Fisher Scientific, Waltham, MA, USA; www.thermo.com, accessed on 10 September 2022) for EA-IRMS analyses. EA-IRMS analyses were performed as previously described [24]. The natural abundances of nitrogen and carbon stable isotopes were expressed in per-mill using the δ-notation:

δ (‰) = 1000 × (R_sample_ − R_standard_)/R_standard_, (2)

where R is the ratio of the heavy to light isotopes in the sample and standards (atmospheric N_2_ for ^15^N/^14^N and Vienna Pee Dee Belemnite for ^13^C/^12^C). A short food frequency questionnaire was administered before randomization, and we used the data to obtain information on the intake of foods known to affect body isotopic composition.

### 2.6. Statistical Analyses

The effects of fish oil supplementation on body weight, plasma FA, proinflammatory cytokines, and δ^15^N and ^13^C were assessed using repeated measure ANOVAs with post hoc comparisons (PROC MIXED)(SAS^®^ OnDemand for Academics). IL-6 and TNF-α data were normalized using a log10(x + 1) transformation for statistical analyses and graphical presentation due to a high level of interindividual variability. Patient characteristics of the fish oil and control groups were compared using *t*-tests and Fisher’s exact test for proportions. Differences between groups in the change in δ^15^N and ^13^C were assessed using the *t*-test. The Shapiro–Wilk test was used to determine if data are normally distributed and when necessary, the non-parametric Mann–Whitney U test was performed instead of the *t*-Test. A partial least squares discriminant analysis (PLS-DA) was performed using R package mixOmics [25]. The PLS-DA model performance was tuned with 10-fold cross-validation. Alpha was set at 0.05 and data are presented as mean ± SEM. The participant flow chart (Figure 2) specifies the samples used for each analysis.

## 3. Results

Characteristics of breast cancer patients along with markers of cancer severity are presented in Table 2. Randomization resulted in similar groups and the 46 patients who completed the study did not differ from the others (Table S2). The only exception was the Scarff–Bloom–Richardson (SBR) grade with a greater proportion of grade 1 in the control group. Nonetheless, there was no significant difference between the fish oil and control groups in the proportions of SBR grades II and III and over 70% of patients in both groups had an SBR grade above I. There was a greater proportion of SBR grade III in patients who complied with the supplementation and in the subgroup on which isotopic analyses were performed (Table S2). Body weight did not change during the trial. Blood samples were obtained 10.3 ± 0.6 days (median = 9) and 97.5 ± 3.1 days (median = 96) after supplementation onset and baseline sampling (Figure 1).

**Figure 3:**
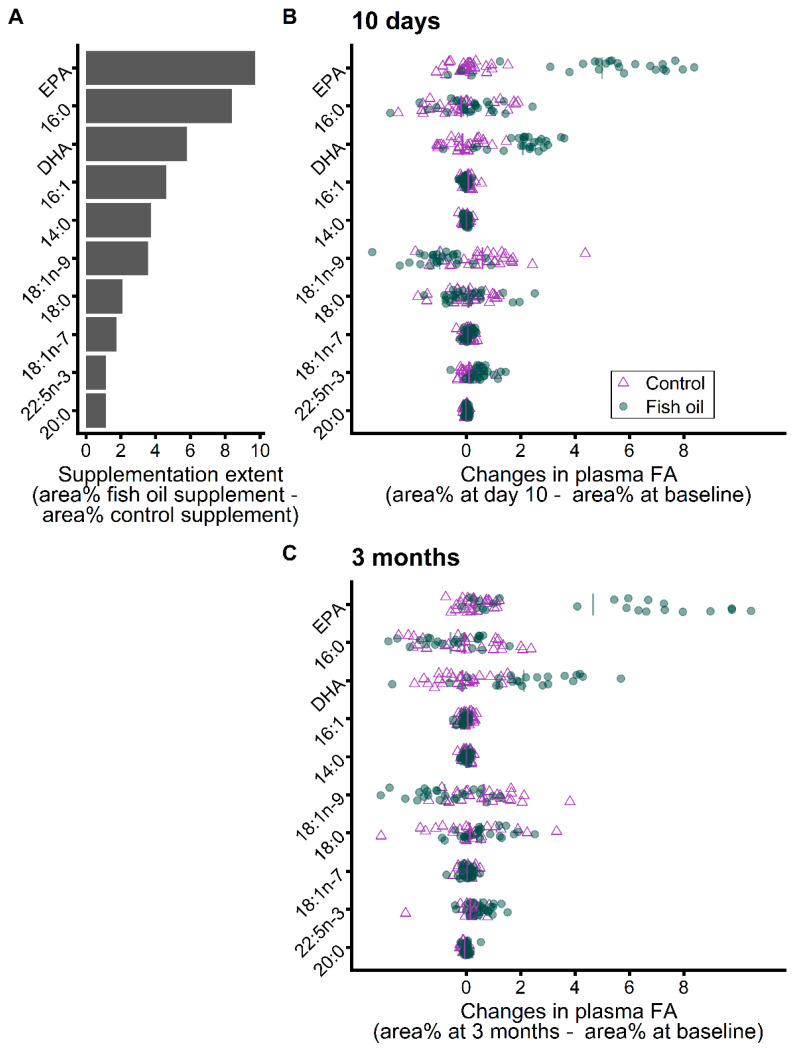
(**A**) Difference between the fatty acid compositions of the fish oil and control supplements (based on fatty acid profiles of supplements (Table S1): fatty acid area% in the fish oil supplement–fatty acid area% in the control supplement). (**B**) Early changes in plasma fatty acid proportions that occurred 10 days after supplementation onset. (**C**) Later changes in plasma fatty acid proportions that occurred 3 months after supplementation onset. Lines represent group averages. Values are sorted according to differences between supplements in fatty acid compositions and only presented for fatty acids with a difference between supplements ˃1 area%. EPA, eicosapentaenoic acid (20:5n-3); DHA, docosahexaenoic acid (22:6n-3).

First, we appreciated the extent of the FA supplementation by calculating the difference between the levels (area%) of each FA in the fish oil and control supplements. This difference between supplement FA profiles is presented in Figure 3A for the FA for which supplements differ by ˃1 area%. EPA (20:5n-3) was the most prevalent FA in the fish oil compared to in the control supplement, followed by 16:0 and DHA (22:6n-3) and 7 other FA were more prevalent by ˃1 area% in the fish oil supplement (Figure 3A). Figure 3B,C present individual changes in plasma FA levels that occurred during the supplementation for these FA that differ by ˃1 area% between supplements. Individual changes in plasma FA levels show that while some FA, and mostly EPA and DHA, increased in line with their intake, some, such as 16:0, remained mostly unchanged, and others, such as 18:1n-9, decreased despite a greater intake (Figure 3), indicating a metabolic response to the fish oil supplementation. However, before investigating metabolic responses, we first had to assess compliance since changes in plasma EPA clearly show a certain degree of non-compliance and its increased prevalence over time. Non-compliance is evidenced by changes in the plasma EPA of individuals in the fish oil supplementation group clustering with those of the control group (Figure 3B,C). We investigated the relationship between reported compliance and plasma FA levels to determine the individuals for whom the metabolic and inflammatory responses could be studied.

### 3.1. Compliance with Fish Oil Supplementation Increases Plasma EPA and DHA

**Figure 4:**
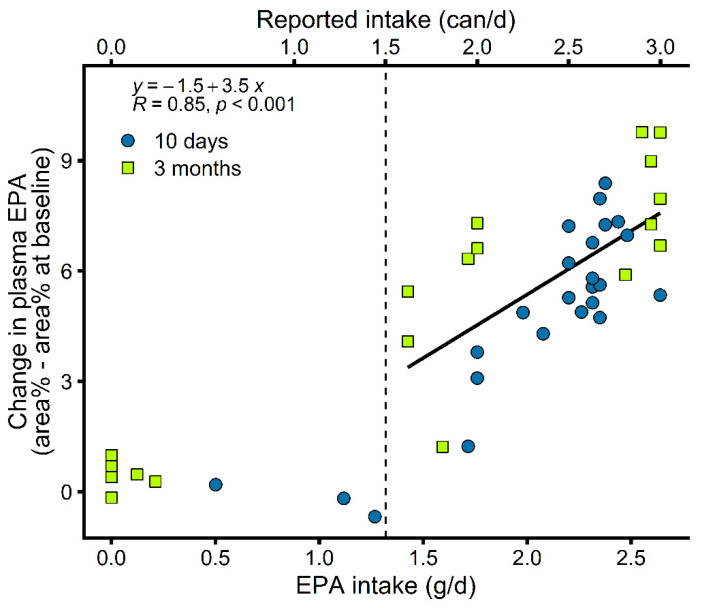
A Plasma EPA dose-response in breast cancer patients supplemented with fish oil was assessed using reported supplement intake. Plasma EPA started increasing at a threshold of approximately 1.3 g of EPA intake per day, corresponding to the intake of 1.5 cans per day of the supplement. We used this reported supplement intake value as a threshold to assess the compliance of both groups (dashed line). EPA, eicosapentaenoic acid (20:5n-3).

**Figure 5:**
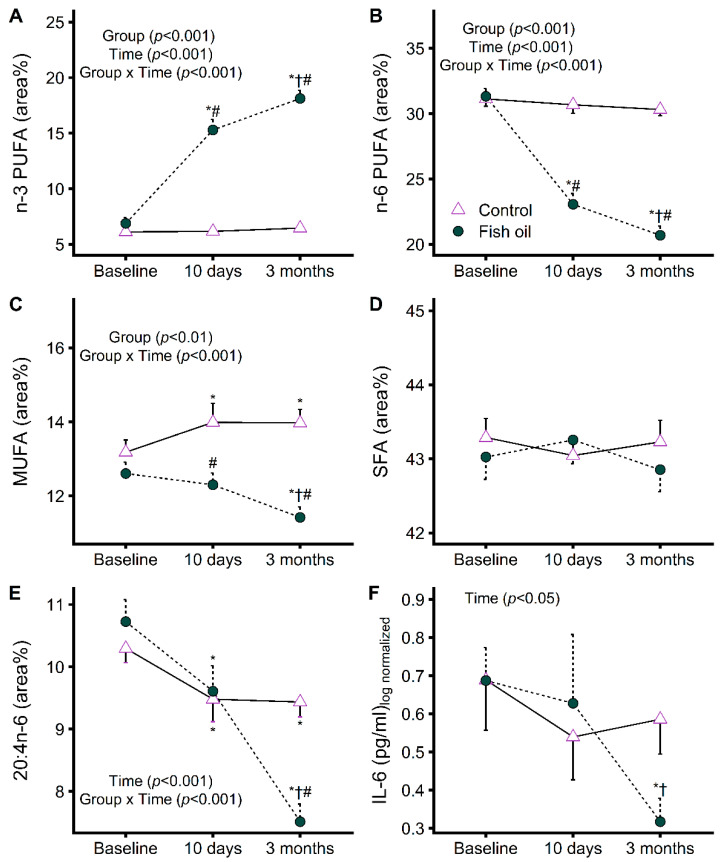
Plasma levels of n-3 (**A**) and n-6 (**B**) polyunsaturated fatty acid(s) (PUFA), monounsaturated fatty acid(s) (MUFA) (**C**), saturated fatty acid(s) (SFA) (**D**), 20:4n-6 (**E**), and interleukin-6 (IL-6) (**F**) in advanced breast cancer patients who complied with fish oil supplementation (*n* = 14) or with a control supplementation (*n* = 17) throughout the trial. * Significant difference with baseline values (*p* < 0.05). † Significant difference with values at 10 days (*p* < 0.05). # Significant difference with control (*p* < 0.05).

EPA and DHA were the only FA with proportions that considerably increased in plasma PL during fish oil supplementation. While a certain degree of non-compliance was clear in the group supplemented with fish oil (Figure 3B,C), it was more challenging to assess compliance of the control group using plasma FA levels. This is because the control supplement (matching caloric and nutrient intake) was mostly richer in medium-chain FA compared to the fish oil supplement (Table S1). Medium-chain FA are metabolized faster than longer chain FA likely because they rely less on proteins for their transports and are not considered to be stored in adipose tissue [26] and we did not analyze their plasma levels. For these reasons, changes in plasma FA induced by the control supplement could be less directly related to compliance. Consequently, we determined a reported intake threshold to assess the compliance of both groups. To do that, we used reported supplement intake from patient diaries of the group taking fish oil to investigate the relationship between compliance and plasma n-3 FA. Plasma EPA of n-3 FA was the most strongly associated with its reported intake (*R* = 0.85 for EPA (Figure 4) and *R* = 0.64 for DHA). This analysis of EPA dose–response confirms a drop in compliance with time during prolonged supplementation and revealed a threshold of around 1.3 g of EPA intake per day from where plasma EPA seems to start increasing with EPA intake (Figure 4). This corresponds to the intake of 1.5 cans per day of the supplement and we used this reported intake value as a threshold to assess the compliance of the control and fish oil groups. Although variability in n-3 PUFA absorption and metabolism cannot be excluded, the strong relationship between the reported supplement intake and EPA levels supports non-compliance in the individuals that were excluded from further analyses. Polymorphisms affecting n-3 PUFA levels are likely more related to the level of variability observed within complying patients (Figure 4). For one time point or the other, reported intake values were missing from the patient diaries of four patients in the fish oil supplementation group, three of whom were considered compliant based on changes in plasma EPA. Moreover, two patients in the fish oil supplementation group likely misreported their supplement intake with no corresponding increases in plasma EPA (Figure 4), which were at levels seen in the control group (Figure 3A,B). Such misreporting, although minimal, could not be detected in the control group due to no FA in the control supplement being a good intake marker. Altogether, these analyses resulted in 31 patients being considered compliant throughout the study (Figure 2) and were used to analyze the effects of the supplementation on inflammatory and metabolic responses (Figure 5).

### 3.2. Impact of Increasing EPA and DHA Levels on Plasma Fatty Acid Profile and Proinflammatory Cytokines

The assessment of compliance allowed us to identify the participants for whom we could investigate the effects of increasing EPA and DHA levels on plasma FA and proinflammatory cytokine kinetics. In line with its high EPA and DHA contents (Table 1), fish oil increased total plasma n-3 PUFA while it decreased total n-6 PUFA as a metabolic consequence (Figure 5A,B). These changes occurred rapidly within 10 days and continued to a smaller extent with prolonged supplementation. MUFA were lowered (Figure 5C), but to a smaller extent than n-6 PUFA, whereas saturated FA remained unchanged (Figure 5D). As opposed to other n-6 PUFA, the inflammatory precursor 20:4n-6 was only lowered compared to the control after prolonged supplementation (Figure 5E), in line with the decrease in IL-6 that followed the same pattern (Figure 5F). TNF-α also followed a similar pattern but with more interindividual variability (Figure S2). These results show that while some circulating FA respond rapidly to fish oil supplementation, it takes longer for inflammation to decrease.

### 3.3. Increasing EPA and DHA Levels Rapidly Impacts Protein Metabolism

**Figure 6:**
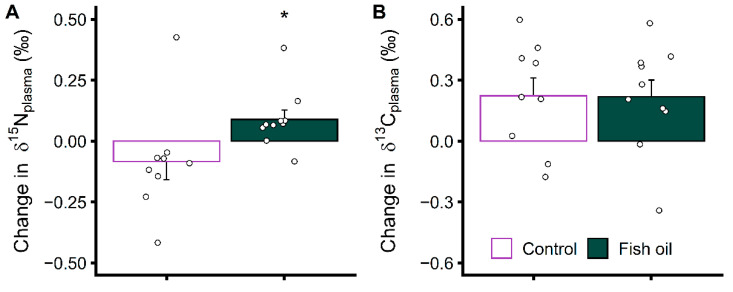
Early changes in plasma δ^15^N (**A**) and δ^13^C (**B**) in a subsample of advanced breast cancer patients who complied with fish oil supplementation (*n* = 10) or with a control supplementation (*n* = 9). Changes in δ^15^N and δ^13^C are the differences between the δ values at 10 days and those at baseline, calculated to normalize the impact of dietary input and isolate the effect of metabolism. The δ^15^N was lower in the supplements (5.3 ‰, Table 1) than in the plasma (Table 3), indicating that the increase in δ^15^N during fish oil supplementation does not reflect the direct incorporation of the supplement’s amino acids into the plasma as this would rather decrease its δ^15^N. While the supplementation replaced part of the usual food intake of the patients, it is unlikely that the supplement substituted foods with a lower δ^15^N in the fish oil group and that it rather substituted foods with a higher δ^15^N in the control group. The δ^13^C was also lower in the supplements than in the plasma suggesting that the increase in δ^13^C in both groups during the supplementation has a metabolic origin. * Significant difference between groups.

We investigated changes in protein metabolism using natural δ^15^N variations, which have been shown to occur during changes in protein and amino acid metabolism [17,18,21]. Plasma isotopic composition is presented in Table 3. The body isotopic composition is also affected by dietary input with no significant baseline difference between groups in δ^15^N and δ^13^C (Table 3) as well as in plasma fatty acid levels (Figure 5, Table S3), suggesting similar diets. The stable body weight throughout the study supports no change in caloric intake. In addition, reported food intake frequencies confirm no difference between groups in food choices affecting body isotopic composition (Figures S3 and S4) but there is a need for a more sophisticated approach to assess dietary variations. Long-term variations in dietary input likely prevented the time by group interaction on plasma δ^15^N to reach statistical significance (*p* = 0.10) (Table 3). Indeed, baseline δ values and those at 10 days of supplementation displayed a good correlation (*R* = 0.81 for δ^15^N and *R* = 0.81 for δ^13^C) while δ values at 3 months were less well correlated with baseline values (*R* = 0.73 for δ^15^N and *R* = 0.34 for δ^13^C). We acknowledge that the small sample size and incomplete knowledge of dietary variations are limitations of this study. To normalize the impact of dietary input and isolate the effect of metabolism, we evaluated the early change in isotopic compositions that occurred during the supplementation (δ_10 days–_δ_baseline_, Figure 6A). This approach estimates δ^15^N variations due to metabolic changes, analogous to variations in values expressed with the capital delta notation (Δ^15^N) in animal models where dietary input is well controlled [17,18]. Values at 3 months were not normalized the same way since it would not account for dietary changes that are more likely to have occurred in the meantime. Our analysis performed on a subsample of compliant patients showed a rapid opposite change in plasma δ^15^N in the group supplemented with fish oil compared to in the control group (*p* < 0.05) (Figure 6A) indicating a rapid impact on protein metabolism.

Then, we used natural δ^13^C variations to further assess the metabolic impact of the supplementation. Our results provide some evidence that the fish oil and control supplements, despite the change in protein metabolism induced by the former, had the same impact on whole-body substrate oxidation. The group supplemented with fish oil displayed the same increase in δ^13^C as the control group (Figure 6B) (Table 3). This further confirms compliance in these patients selected for isotopic analyses and suggests that changes in protein metabolism during fish oil supplementation are not related to major changes in whole-body substrate oxidation. We hypothesize that the control and fish oil supplements, due to their identical macronutrient compositions (Table 1), resulted in similar changes in the substrate mix in circulation that fuel tissue oxidative metabolism, explaining the similar changes in bulk plasma δ^13^C. A lack of effect on whole-body substrate oxidation of n-3 PUFA supplementation is backed by a recent indirect calorimetry study [27] but not by other studies [28,29,30], emphasizing that this response may be polyfactorial. Here, only EPA and DHA considerably increased in plasma PL during fish oil supplementation while there was a clear metabolic impact on other FA such as 18:1n-9 and the n-6 PUFA (Figure 3 and Figure 5) that decreased despite similar or higher intakes, but bulk plasma δ^13^C was not sensitive enough to detect changes in FA levels and metabolism. Deciphering specifically which FA are metabolized differently may help bring further clues into the mechanism behind the change in protein metabolism that we highlighted using natural δ^15^N variations.

### 3.4. During EPA and DHA Supplementation, More Fatty Acids Reflect Metabolic Regulations Than Their Intake Levels

To pinpoint specifically which FA are affected by metabolic regulations, a more granular analysis of plasma FA profile was performed (Table S3). Individual FA kinetics throughout the supplementation show that other FA than those more prevalent in the fish oil supplement were affected by the supplementation. All major plasma n-3 PUFA were increased except the nutritionally essential 18:3n-3. Although supplied in similar amounts to both groups, plasma 18:3n-3 was unaffected by fish oil supplementation and increased in the control group. Although MUFA were more prevalent in the fish oil supplement (Table S1), circulating levels decreased during fish oil supplementation (Figure 5C). This decrease in plasma MUFA was mostly related to 18:1n-9 (Table S3). Levels of saturated FA remained stable, only plasma 17:0 and 21:0 were affected by fish oil supplementation (Table S3). Altogether, these data combined with supplement FA compositions (Table S1) show that changes in plasma FA levels reflect FA intake for some FA but not for others, indicating a metabolic impact of the supplementation.

**Figure 7:**
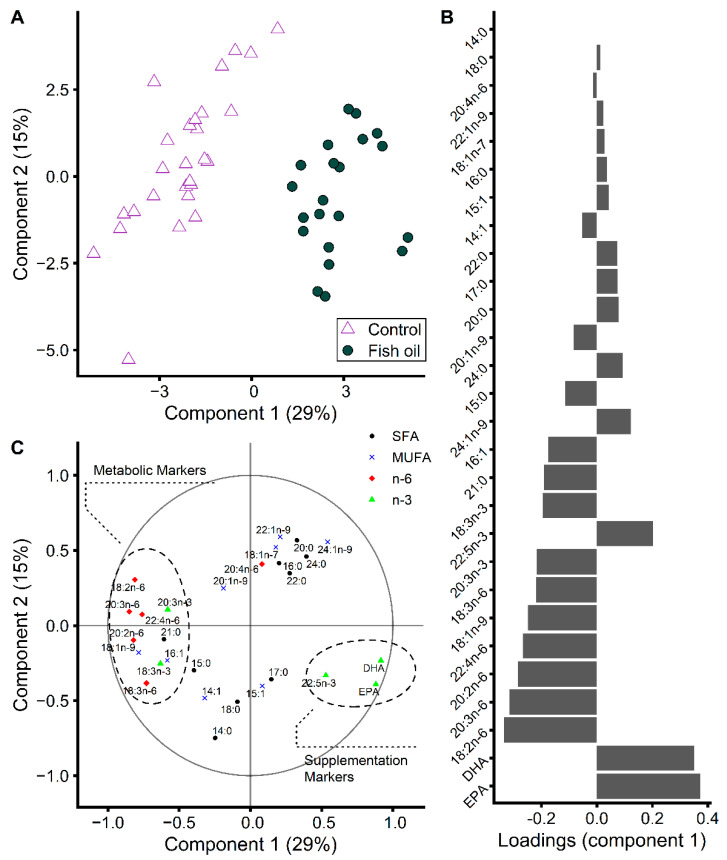
A partial least squares discriminant analysis was performed to further assess, explore, and highlight how changes in fatty acid levels relate to intake and metabolic differences between groups of advanced breast cancer patients who complied with fish oil supplementation (*n* = 21) or with a control supplementation (*n* = 27) during the first 10 days of the trial. (**A**) Score plot. (**B**) Variable correlation plot. (C) Loadings on the first component. Input variables are the differences between fatty acid area% at 10 days and baseline. EPA, eicosapentaenoic acid (20:5n-3); DHA, docosahexaenoic acid (22:6n-3); SFA, saturated fatty acids; MUFA, monounsaturated fatty acids.

To further assess, explore, and highlight how changes in FA levels relate to fish oil intake and its metabolic impact, we used a PLS-DA (Figure 7). Others previously used PLS-DA or principal component analysis before and after dietary FA interventions to appreciate compliance [31,32]. Here, we focused on early changes in FA proportions that occurred within 10 days of supplementation (Figure 2). The reasons were to avoid any confounding effect of chemotherapy, to benefit from a bigger sample of complying patients, and to highlight the FA that respond to fish oil supplementation, irrespectively of supplementation length. Using this approach, we were able to label some FA as markers of the supplementation and others as markers of the metabolic impact. For each FA rapidly displaying changed levels during the supplementation, we evaluated the prevalence of the FA in the fish oil supplement compared to in the control supplement. We found that only 3 FA, the n-3 PUFA EPA, DHA, and 22:5n-3, were more prevalent in the fish oil supplement by ˃ 1 area% (Table S1) and had a rapid increase in their plasma levels upon the supplementation (Table S3). These 3 FA clustered in the PLS-DA correlation plot and were labeled as supplementation markers (Figure 7C). EPA and DHA were the FA with the highest importance in the first component of the PLD-DA model (Figure 7B). While 17:0 displayed slightly higher plasma levels upon fish oil supplementation (Table S3), it was present in the same amount in the fish oil and control supplements with only a 0.16 area% difference between supplements (Table S1) and was consequently not labeled as a supplementation marker. While being more prevalent in the fish oil supplement (1.75 area% difference between supplements), plasma 18:1n-7 did not increase (Table S3) and was consequently not considered as a supplementation marker. Ten FA were labeled as metabolic markers and clustered to the other side of the PLS-DA correlation plot (Figure 7C). All major n-6 FA were present in similar quantities in the fish oil and control supplements (<1 area% difference, Table S1) and lowered in plasma after 10 days of fish oil supplementation (Table S3) except 20:4n-6. 20:4n-6 decreased only compared to control after long-term supplementation (Figure 5) and had modest importance in the first component of our PLS-DA model based on short-term changes (Figure 7B). All n-6 FA were consequently labeled as metabolic markers except 20:4n-6 (Figure 7C). The n-3 FA 18-3n-3 and 20:3n-3 were labeled as metabolic markers since they were present in similar amounts in the fish oil and control supplements (Table S1) while displaying changed plasma levels after the supplementation (Table S3). 16:1 and 21:0 were also labeled as metabolic markers (Figure 7C). The fish oil supplement was enriched in the former (Table S1) while plasma levels slightly decreased. 21:0 decreased with fish oil supplementation (Table S3) but was present in similar quantities in the fish oil and control supplements (Table S1). Tuning model performance using cross-validation confirms the pertinence of our labeling of FA as intake and metabolic markers. The classification error rate was quasi-null for a one-component model using as input variables the 12 FA labeled in Figure 7C. Altogether, these analyses show that more FA are affected by metabolic regulations than by differences in their intake during fish oil supplementation. Our labeling of FA as either supplementation or metabolic markers provides a good rule of thumb to assess supplementation impact irrespectively of supplementation length. Care should be taken when considering other FA to assess supplementation impact, especially the inflammatory precursor 20:4n-6 since its response is more time-dependent.

We then sought to investigate how changes in FA metabolism induced by fish oil supplementation relate to desaturase enzyme activities. Indices of enzymatic activity were determined using ratios between precursors and products. The rationale behind this approach is that concentrations of precursors and products of desaturation reactions depend on fluxes through these reactions and reflect enzymatic activity rather than the intake of the FA. This excluded EPA, DHA, and 22:5n-3 from being used due to increased plasma area% after supplementation with fish oil, which is rich in these n-3 PUFA (Table S1). Of the FA used for assessing indices of enzymatic activity, 18:0 and 18:1n-9 were clearly in excess in the fish oil compared to in the control supplement (Figure 3 and Table S1), but their plasma proportions remained unchanged or decreased during the supplementation (Table S3), supporting that these FA reflect metabolic activity rather than their intake. Our analyses suggest that fish oil supplementation decreased the activity of the Δ6- and-9 desaturases and increased the activity of the Δ5-desaturase (Figure S5).

## 4. Discussion

The results of this trial show an uncoupling between changes in inflammation and protein metabolism during n-3 fatty acid supplementation. Whereas n-3 PUFA are effective at lowering proinflammatory cytokines in advanced breast cancer patients (Figure 5), protein metabolism is affected way faster (Figure 6). Measurement of natural ^15^N abundance allowed us to identify changes in protein and amino acid metabolism occurring within 10 days of fish oil supplementation, while inflammation was only later lowered. We documented a considerable decrease with time in the percentage of patients that complied with the supplementation, making compliance assessment crucial for the kinetic analysis of the metabolic and inflammatory responses. Our results show that natural ^13^C abundance could be useful for the assessment of compliance and metabolic impact. The change in proinflammatory cytokines was concomitant to a delayed decrease in the plasma level of the inflammatory precursor 20:4n-6. This is contrasting with the rapid changes in the proportion of many FA that occurred during the supplementation, including many n-6 FA. By using a PLS-DA in conjunction with FA contents in the fish oil and control supplements, we highlighted the FA that are rapid direct markers of the supplementation and those that rapidly reflect its metabolic impact. Finally, we attempted to relate these changes in FA levels to the activity of desaturase enzymes.

There is no clear knowledge of the dose of n-3 PUFA required for them to exert their effect [1]. It has been suggested that the threshold for an anti-inflammatory effect of EPA could lie between 1.35 and 2.7 g per day of intake [7,33]. Here, compliance with fish oil supplementation led to the intake of 1.3 to 2.6 g of EPA per day (Figure 4) and 0.8 to 1.6 g of DHA per day. At this dose, proinflammatory cytokines remained initially unchanged but were reduced after 3 months (Figure 5F and Figure S2). Whereas it could be questioned whether chemotherapy, initiated after 10 days of supplementation, had a role to play in the long-term modulation of proinflammatory cytokines by n-3 PUFA, cytokines remained at the baseline level in the control group during chemotherapy.

We found a rapid change in protein metabolism unrelated to a later decrease in proinflammatory cytokines in advanced breast cancer patients supplemented with n-3 PUFA. The question remains as to what specific change in protein metabolism the natural δ^15^N variations measured here reflect. It was shown that δ^15^N in muscles, compared to in splanchnic organs, such as the liver, behave opposingly with changes in protein and amino acid metabolism [17,21]. The nitrogen in the plasma pool is what was analyzed here. It is a mix of the nitrogen found in free amino acids, proteins, and other nitrogenous compounds, such as urea. While plasma-free amino acids come from both splanchnic organs consequently to protein turnover and amino acid metabolism, they also originate from the turnover and metabolism of peripheral organs, such as muscles. This is less the case for plasma proteins since albumin and other liver-synthesized proteins represent a substantial proportion of plasma proteins. In the fasted state, net circulating amino acid flux is from peripheral towards splanchnic organs, especially from the muscles [34,35], but the breast cancer patients participating in this study were not in the fasted state at the time of blood sampling. Considering all this, it is difficult to pinpoint the tissues involved in the metabolic response observed. Still, the increase in plasma δ^15^N in metastatic breast cancer patients during n-3 PUFA supplementation (Figure 6) can be argued to reflect both an increased amino acid catabolism in the liver and opposingly an increased amino acid anabolism in the muscles or their combination [17,21]. The magnitude of the effect, at least in some subjects, might suggest a combined effect. It must also be noted that, in healthy individuals, the turnover time of plasma albumin is about 25 days with a half-life of 17.3 days [36] and the turnover time of muscle proteins is longer [37]. This indicates that in the case of the rapid effect observed here within 10 days of supplementation, liver amino acid metabolism would impact δ^15^N more than muscle protein metabolism. Moreover, it cannot be disregarded that the plasma pool contains a mix of free amino acids of different δ^15^N [38] and that changes in their proportions, due to changes in tissue inward and outward fluxes, will affect the δ^15^N of the whole pool. The capacity of changes in plasma-free amino acid concentrations to affect the δ^15^N of the whole pool is, however, questionable from a quantitative point of view given the low concentration of free amino acids compared to proteins and the small, expected difference in δ^15^N between them. Still, it can be speculated that changes in plasma δ^15^N are, at least partly, affected by changes in plasma concentration of one or several amino acids due to a shift in muscle metabolism. There is evidence that n-3 PUFA supplementation can increase plasma concentration of branched-chain amino acids by reducing their degradation [39,40] and that it can enhance muscle protein anabolism [6]. These findings are in line with the interpretation that the increased plasma δ^15^N observed after n-3 PUFA supplementation (Figure 6) reflects a reduced amino acid catabolism in muscles. Deciphering liver and muscle metabolism may be achieved through compound-specific isotope analysis. Separating plasma-free amino acids from plasma proteins and analyzing the δ^15^N of individual amino acids in both fractions using gas chromatography isotope ratio mass spectrometry [17,38], appears promising.

The effects of n-3 PUFA on protein metabolism have been demonstrated in skeletal muscles [6] and associated with FA desaturation [41]. However, much less is known about the n-3 PUFA effect on hepatic protein metabolism. Moreover, there is not so much research on the role of the liver in the relation between inflammation and protein wasting during cancer [42]. Since the liver is the primary site of PL synthesis [43] and Δ5-and-6 desaturases expression can be high there compared to in other tissues [44], changes in the indices of the enzymatic activity assessed from plasma PL FA ratios (Figure S5) could mostly reflect hepatic activity and relate to hepatic protein and amino acid metabolism. N-3 PUFA intake has been linked with Δ5-,6-, and-9 desaturase activities with the involvement of both competitive and feedback mechanisms [5,45,46,47,48]. Here, the shorter chain n-6 and n-3 PUFA were provided in similar amounts to the fish oil and control groups resulting in no difference in their competition for desaturation. However, fish oil is rich in the longer chain EPA and DHA, supporting a feedback inhibition of the Δ6-, and-9 desaturases (Figure S5). The more surprising observed increase in Δ5-desaturase activity index (Figure S5), which has already been reported by others using the same technique [49,50,51], could suggest a compensatory response that would be aimed at holding back the change in n-6 PUFA. A Compensatory stimulation of Δ5-desaturase has been observed in mice with reduced Δ6-desaturase expression [52]. Additionally, a recent study found opposite associations between fatty acid desaturase gene variants and Δ5-and Δ6- desaturase expressions [53]. Still, our results also raise more questions about assessing FA fluxes with precursors to product ratios since metabolic fluxes can theoretically change differently than concentrations. Moreover, FA levels here are not reported as concentrations, but as chromatographic proportions (area%, equation 1). More work is required to determine the validity of this technique to assess enzymatic activity and the relation between FA desaturation and protein and amino acid fluxes. Measuring the natural isotopic abundance of carbon in individual FA could yield further information on enzymatic activity.

Early detection is important in clinical practice to adjust therapeutic interventions. Our results show that both compliance and supplementation metabolic impact can be rapidly assessed using changes in individual FA proportions (Figure 7), while care should be taken about the inflammatory precursor 20:4n-6 that takes longer to react. We expect the combined measurement of individual FA levels and their natural ^13^C isotopic abundance to bring additional layers of information on both compliance and metabolic regulations.

## 5. Conclusions

Strengthening the body of clinical evidence on the effect of n-3 PUFA will require good tools to assess compliance and the metabolic impact. Natural variations of ^15^N abundance were measured during this clinical trial and our results advocate for a model where n-3 PUFA impact protein metabolism independently of systemic inflammation that is only later reduced. This could have implications for cachexia during which inflammation is thought to play a central role in body protein wasting [42] and for which further investigations are required. Future studies should include assessments of tissue-specific body composition changes, better assess dietary intake variations during the nutritional supplementation, and include compound-specific isotope analyses.

## Figures and Tables

**Table 1:** Nutritional and isotopic compositions of the fish oil and control supplements.

	Control Supplement	Fish Oil Supplement
Nutritional composition	per 200 mL can	
Energy	300 Kcal	300 Kcal
Proteins	15 g	15 g
Carbohydrates	36 g	36 g
Lipids	12 g	12 g
Saturated FA	6.6 g	3 g
Monounsaturated FA	3.8 g	5 g
PUFA	1.1 g	4 g
18:2n-6	1 g	1 g
18:3n-3	0.5 g	0.5 g
EPA	0 g	0.88 g
DHA	0 g	0.52 g
Isotopic composition		
δ^15^N	5.3‰	5.3‰
δ^13^C	−25.4‰	−24.6‰

FA, fatty acids; PUFA, polyunsaturated fatty acids; EPA, eicosapentaenoic acid (20:5n-3); DHA, docosahexaenoic acid (22:6n-3). The nutritional composition of the supplements was provided by the manufacturer. We performed a detailed analysis of the fatty acid profile of the supplements (Table S1).

**Table 2:** Patient characteristics.

	Control (*n* = 32)	Fish oil (*n* = 31)	*p*
Age (y)	57.0 ± 2.1	60.6 ± 2.1	0.23
Body weight (kg)	69.2 ± 2.2	66.3 ± 1.9	0.32
BMI	26.6 ± 0.8	25.5 ± 0.7	0.31
Menopausal status			
Non-menopausal	4/29 (14%)	4/30 (13%)	1.0
Peri-menopausal	3/29 (10%)	2/30 (7%)	0.67
Menopausal	22/29 (76%)	24/30 (80%)	0.76
Not available	3	1	
Disease severity at diagnosis *			
Node status			
N0	7/27 (26%)	5/28 (18%)	0.53
N1	15/27 (56%)	16/28 (57%)	1.0
N2	3/27 (11%)	2/28 (7%)	0.67
N3	2/27 (7%)	5/28 (18%)	0.42
Not available	5	3	
SBR grade			
I	9/32 (28%)	2/28 (7%)	<0.05
II	18/32 (56%)	17/28 (61%)	0.80
III	5/32 (16%)	9/28 (32%)	0.22
Not available	0	3	
Metastases at inclusion			
Number of metastases ^†^	2.2 ± 0.2	2.3 ± 0.2	0.69
Pleuropulmonary region	10/32 (31%)	10/31 (32%)	1.0
Liver	16/32 (50%)	16/31 (52%)	1.0
Node	15/32 (47%)	8/31 (26%)	0.12
Bone	24/32 (75%)	25/31 (81%)	0.76

* Clinical markers obtained at the time of breast cancer diagnosis and expressed according to tumor- node-metastasis classification. Inclusion in the study was on average 10 days before chemotherapy onset (Figure 1) and chemotherapy could start long after diagnosis depending on the delay for metastasis development and the need for chemotherapy. ^†^ Sum of the numbers of bone and visceral affections. BMI, body mass index; SBR, Scarff–Bloom–Richardson.

**Table 3:** Plasma δ^15^N and δ^13^C in a subsample of advanced breast cancer patients who complied with fish oil supplementation (*n* = 10) or with a control supplementation (*n* = 9).

	Baseline		10 Days		3 Months		*p*		
	Control	Fish Oil	Control	Fish Oil	Control	Fish Oil	Group	Time	Group × Time
δ^15^N	9.2 ± 0.1	9.0 ± 0.1	9.1 ± 0.1	9.1 ± 0.1	9.1 ± 0.1	9.1 ± 0.1	0.75	0.98	0.10
δ^13^C	−22.2 ± 0.2	−22.1 ± 0.1	−22.0 ± 0.2	−21.9 ± 0.1	−21.7 ± 0.2 *^,†^	−21.7 ± 0.1 *	0.55	<0.001	0.67

* Significant difference with pre-supplementation values (*p* < 0.05). ^†^ Significant difference with values at 10 days (*p* < 0.05).

## Data Availability

The data are available from the corresponding author upon reasonable request.

## Supplementary Materials

The following supporting information can be downloaded at: https://www.mdpi.com/article/10.3390/metabo12100899/s1, Figure S1: Typical chromatograms of plasma phospholipid fatty acid methyl esters for a patient that complied with the control supplementation (A) and for one that complied with the fish oil supplementation (B); Figure S2: Plasma tumor necrosis factor-alpha (TNF) in advanced breast cancer patients who complied with fish oil supplementation (*n* = 14) or with a control supplementation (*n* = 17) throughout the trial; Figure S3: Reported frequencies of meat and fish intake (A) and indices of animal-based (B) and plant-based (C) food intakes in the subsample of advanced breast cancer patients used for isotopic analyses (fish oil supplementation: *n* = 10, control supplementation; *n* = 9); Figure S4: Association between animal-based food consumption index and baseline plasma δ^13^C in the subsample of advanced breast cancer patients used for isotopic analyses (*n* = 19); Figure S5: Indices of enzymatic activity in advanced breast cancer patients who complied with fish oil supplementation (*n* = 14) or with a control supplementation (*n* = 17) throughout the trial. Plasma ratios between precursors and products were used to estimate the activity of (A) Stearoyl-CoA desaturase 1 (SCD1, Δ9-desaturase) (18:1n-9/18:0), (B) fatty acid desaturase 2 (FADS2, Δ6-desaturase) (18:3n-6/18:2n-6), (C) fatty acid desaturase 1 (FADS1, Δ5-desaturase) (20:4n-6/20:3n-6); Table S1: Fatty acid compositions of the control and fish oil supplements (area%); Table S2: Characteristics of participants who completed the study, of those who complied with the supplementation and of the subgroup for which isotopic analyses were performed; Table S3: Effect of fish oil supplementation on plasma fatty acids (%area).
