# Supplementary material for: N-3 Fatty Acid Supplementation Impacts Protein Metabolism Faster Than it Lowers Proinflammatory Cytokines in Advanced Breast Cancer Patients: Natural 15N/14N Variations during a Clinical Trial
Source: Metabolites. 2022 Sep 24;12(10):899. doi: 10.3390/metabo12100899 (PMC9609900; doi:10.3390/metabo12100899)
Supplement: Supplementary file 1 [file metabolites-12-00899-s001.zip › metabolites-1863769-supplementary.pdf]

Table S1. Fatty acid compositions of the control and fish oil supplements (area%).

	Control	Fish oil
Saturated		
8:0	29.49 ±5.31	2.86 ±1.29
10:0	27.17 ±0.35	3.17 ±0.40
12:0	0.11 ±0.00	0.07 ±0.00
14:0	0.05 ±0.00	3.80 ±0.39
15:0	0.01 ± 0.00	0.30 ±0.02
16:0	2.19 ±0.42	10.58 ±0.25
17:0	0.03 ±0.01	0.28 ±0.08
18:0	0.67 ±0.13	2.76 ±0.40
20:0	0.25 ±0.02	1.38 ±0.10
21:0	0.00 ±0.00	0.02 ±0.00
22:0	0.12 ±0.01	0.15 ±0.02
24:0	0.04 ±0.03	0.12 ±0.08
Σ	60.13	25.49
Monounsaturated		
14:1	0.02 ±0.03	0.13 ±0.03
15:1	0.01 ±0.00	0.05 ±0.02
16:1	0.10 ±0.02	4.72 ±0.32
18:1n-9	25.02 ±3.06	28.58 ±1.22
18:1n-7	1.15 ±0.11	2.90 ±0.21
20:1n-9	0.48 ±0.01	0.77 ±0.02
22:1n-9	0.06 ±0.02	0.18 ±0.09
24:1n-9	0.06 ±0.00	0.22 ±0.02
Σ	26.90	37.55
PUFA		
n-6		
18:2n-6	8.02 ±1.01	7.61 ±0.54
18:3n-6	0.02 ±0.00	0.17 ±0.02
20:2n-6	0.12 ±0.15	0.11 ±0.02
20:3n-6	0.00 ±0.00	0.08 ±0.02
20:4n-6	0.00 ±0.00	0.62 ±0.11
22:4n-6	0.01 ±0.01	0.07 ±0.01
Σ	8.17	8.66
n-3		
18:3n-3	3.46 ±0.85	3.35 ±0.24
20:3n-3	0.00 ±0.00	0.04 ±0.01
20:5n-3	0.01 ±0.01	9.73 ±0.42
22:5n-3	0.00 ±0.00	1.14 ±0.09

22:6n-3	0.02 ±0.04	5.83 ±0.64
Σ	3.49	20.09
n-6/n-3	2.34	0.43

Data are the means of 3 replicates ±SD. PUFA, polyunsaturated fatty acids.

Table S2. Characteristics of participants who completed the trial, of those who complied with the supplementation and of the subgroup for which isotopic analyses were performed.

	Trial completion			Compliance			Isotopic analyses		
	Completed the trial (n = 46)	Others (n = 17)	*P*	Compliers (n = 31)	Others (n = 32)	*P*	Isotopic analyses (n = 19)	Others (n = 44)	*P*
Age (y)	57.9 ±1.6	61.3 ±3.3	.31	56.9 ±2.0	60.6 ±2.2	.22	57.6 ±2.8	59.3 ±1.8	.59
Body weight (kg)	68.1 ±1.6	67.0 ±3.5	.76	67.5 ±1.9	68.2 ±2.2	.83	66.4 ±2.2	68.5 ±1.9	.50
BMI	25.7 ±0.6	27.0 ±1.4	.32	25.3 ±0.7	26.8 ±0.8	.18	25.1 ±0.9	26.4 ±0.7	.27
Menopausal status									
Non-menopausal	6/43 (14%)	2/16 (13%)	1.0	3/28 (11%)	5/31 (16%)	.71	1/16 (6%)	7/43 (16%)	.43
Peri-menopausal	4/43 (9%)	1/16 (6%)	1.0	2/28 (7%)	3/31 (10%)	1.0	1/16 (6%)	4/43 (9%)	1.0
Menopausal	33/43 (77%)	13/16 (81%)	1.0	23/28 (82%)	23/31 (74%)	.54	14/16 (88%)	32/43 (74%)	.48
Not available	3	1		3	1		3	1	
Disease severity at diagnosis*									
Node status									
N0	9/41 (22%)	3/14 (21%)	1.0	6/29 (21%)	6/26 (23%)	1.0	3/18 (17%)	9/37 (24%)	.73
N1	24/41 (59%)	7/14 (50%)	.76	18/29 (62%)	13/26 (50%)	.42	10/18 (56%)	21/37 (57%)	1.0
N2	4/41 (10%)	1/14 (7%)	1.0	2/29 (7%)	3/26 (12%)	.66	2/18 (11%)	3/37 (8%)	1.0
N3	4/41 (10%)	3/14 (21%)	.35	3/29 (10%)	4/26 (15%)	.70	3/18 (17%)	4/37 (11%)	.67
Not available	5	3		2	6		1	7	
SBR grade									

I	7/45 (16%)	4/15 (27%)	.44	3/31 (10%)	8/29 (28%)	.10	2/19 (11%)	9/41 (22%)	.48
II	26/45 (58%)	9/15 (60%)	1.0	17/31 (55%)	18/29 (62%)	.61	8/19 (42%)	27/41 (66%)	.10
III	12/45 (27%)	2/15 (13%)	.48	11/31 (35%)	3/29 (10%)	<.05	9/19 (47%)	5/41 (12%)	<.01
Not Available	1	2		0	3		0	3	
Metastases at inclusion									
Number of metastases†	2.3 ±0.1	2.2 ±0.3	.77	2.1 ±0.2	2.4 ±0.2	.18	2.3 ±0.2	2.2 ±0.2	.69
Pleuropulmonary region	15/46 (33%)	5/17 (29%)	1.0	10/31 (32%)	10/32 (31%)	1.0	5/19 (26%)	15/44 (34%)	.77
Liver	25/46 (54%)	7/17 (41%)	.40	14/31 (45%)	18/32 (56%)	.45	11/19 (58%)	21/44 (48%)	.59
Node	16/46 (35%)	7/17 (41%)	.77	10/31 (32%)	13/32 (41%)	.60	5/19 (26%)	18/44 (41%)	.39
Bone	38/46 (83%)	11/17 (65%)	.17	24/31 (77%)	25/32 (78%)	1.0	17/19 (89%)	32/44 (73%)	.19

*Clinical markers obtained at the time of breast cancer diagnosis and expressed according to tumor-node-metastasis classification. Inclusion in the study was on average 10 days before chemotherapy onset (Figure 1) and chemotherapy could start long after diagnosis depending on the delay for metastasis development and the need for chemotherapy. †Sum of the numbers of bone and visceral affections. BMI, Body mass index; SBR, Scarff-Bloom-Richardson.

Table S3. Effect of fish oil supplementation on plasma fatty acids (%area).

	Baseline				10 days (10.3 ±0.6 days)				3 months (97.5 ±3.1 days)				*P* Group×Time
	Control (*n* = 17)		Fish oil (*n* = 14)		Control (*n* = 17)		Fish oil (*n* = 14)		Control (*n* = 17)		Fish oil (*n* = 14)		
	Mean	SEM	Mean	SEM	Mean	SEM	Mean	SEM	Mean	SEM	Mean	SEM	
Saturated													
14:0	0.39	0.03	0.38	0.02	0.38	0.02	0.39	0.02	0.40	0.03	0.41	0.02	
15:0	0.19	0.01	0.22	0.01	0.20	0.01	0.20	0.01	0.20	0.01	0.21	0.01	
16:0	26.44	0.29	26.02	0.24	26.16	0.23	26.38	0.37	26.44	0.22	25.47[†#]	0.29	<.05
17:0	0.33	0.01	0.38	0.02	0.35	0.01	0.40[#]	0.02	0.34	0.01	0.43[*†#]	0.02	<.05
18:0	12.41	0.36	12.48	0.27	12.57	0.37	12.34	0.21	12.63	0.26	12.94	0.25	
20:0	0.63	0.03	0.58	0.03	0.63	0.02	0.60	0.03	0.56[*†]	0.02	0.59	0.02	<.05
21:0	0.19	0.02	0.19	0.02	0.17	0.02	0.12[*]	0.01	0.17	0.02	0.11[*]	0.01	<.01
22:0	1.54	0.05	1.55	0.06	1.50	0.03	1.57	0.06	1.45	0.05	1.47	0.04	
24:0	1.17	0.05	1.23	0.06	1.10	0.04	1.25[#]	0.05	1.04[*]	0.05	1.20[#]	0.03	
Σ	43.29	0.26	43.02	0.30	43.05	0.26	43.26	0.32	43.23	0.29	42.85	0.30	
Monounsaturated													
14:1	0.01	0.00	0.01	0.00	0.01	0.00	0.00	0.00	0.01	0.00	0.01	0.00	
15:1	0.05	0.01	0.04	0.01	0.04	0.00	0.04	0.01	0.05	0.01	0.04	0.01	
16:1	0.57	0.03	0.54	0.03	0.64	0.05	0.50[#]	0.02	0.63	0.05	0.44[#]	0.02	<.05
18:1n-9	8.63	0.22	8.27	0.25	9.17	0.37	7.50[*#]	0.27	9.40[*]	0.29	6.92[*#]	0.23	<.001
18:1n-7	1.24	0.06	1.30	0.06	1.34	0.07	1.40	0.07	1.32	0.07	1.36	0.05	
20:1n-9	0.14	0.01	0.14	0.01	0.15	0.01	0.12[#]	0.01	0.14	0.01	0.13	0.01	
22:1n-9	0.09	0.01	0.08	0.01	0.09	0.01	0.09	0.01	0.08	0.01	0.07	0.01	

24:1n-9	2.44	0.18	2.24	0.19	2.54	0.20	2.63	0.20	2.34	0.12	2.44	0.22	
Σ	13.18	0.33	12.61	0.30	13.99*	0.51	12.30#	0.32	13.97*	0.37	11.42*†#	0.28	<.001
PUFA													
n-6													
18:2n-6	17.15	0.65	16.90	0.76	17.27	0.79	11.13*#	0.83	16.40	0.61	11.34*#	0.70	<.001
18:3n-6	0.10	0.01	0.10	0.01	0.11	0.01	0.05*#	0.00	0.11	0.01	0.04*#	0.00	<.001
20:2n-6	0.46	0.02	0.48	0.02	0.51	0.02	0.30*#	0.02	0.61*†	0.03	0.29*#	0.02	<.001
20:3n-6	2.73	0.18	2.74	0.16	2.93	0.13	1.73*#	0.13	3.37*†	0.11	1.36*†#	0.09	<.001
20:4n-6	10.30	0.23	10.73	0.35	9.48*	0.36	9.61*	0.41	9.44*	0.24	7.51*†#	0.28	<.001
22:4n-6	0.39	0.02	0.38	0.02	0.37	0.03	0.24*#	0.01	0.39	0.01	0.15*†#	0.01	<.001
Σ	31.12	0.56	31.32	0.60	30.68	0.66	23.06*#	0.82	30.31	0.48	20.70*†#	0.69	<.001
n-3													
18:3n-3	0.17	0.02	0.18	0.02	0.26*	0.02	0.17#	0.01	0.30*	0.03	0.17#	0.01	<.001
20:3n-3	0.06	0.00	0.07	0.00	0.06	0.00	0.06*	0.00	0.07*	0.00	0.05*#	0.00	<.001
20:5n-3	0.97	0.18	1.34	0.20	0.97	0.11	6.88*#	0.59	1.26	0.14	8.66*†#	0.57	<.001
22:5n-3	1.22	0.15	1.25	0.13	1.22	0.19	1.75*#	0.16	1.28	0.11	2.00*#	0.10	<.05
22:6n-3	3.80	0.22	4.05	0.28	3.65	0.19	6.44*#	0.31	3.55	0.25	7.24*†#	0.29	<.001
Σ	6.12	0.40	6.89	0.52	6.17	0.34	15.30a*#	0.93	6.46	0.37	18.12*†#	0.74	<.001
n-6/n-3	5.42	0.34	4.94	0.44	5.27	0.36	1.64*#	0.19	5.00	0.37	1.19*#	0.10	<.001

*Significant difference with baseline values ($p < 0.05$). †Significant difference with values at 10 days ($p < 0.05$). #Significant difference with control ($p < 0.05$). PUFA, polyunsaturated fatty acids.

Figure S1

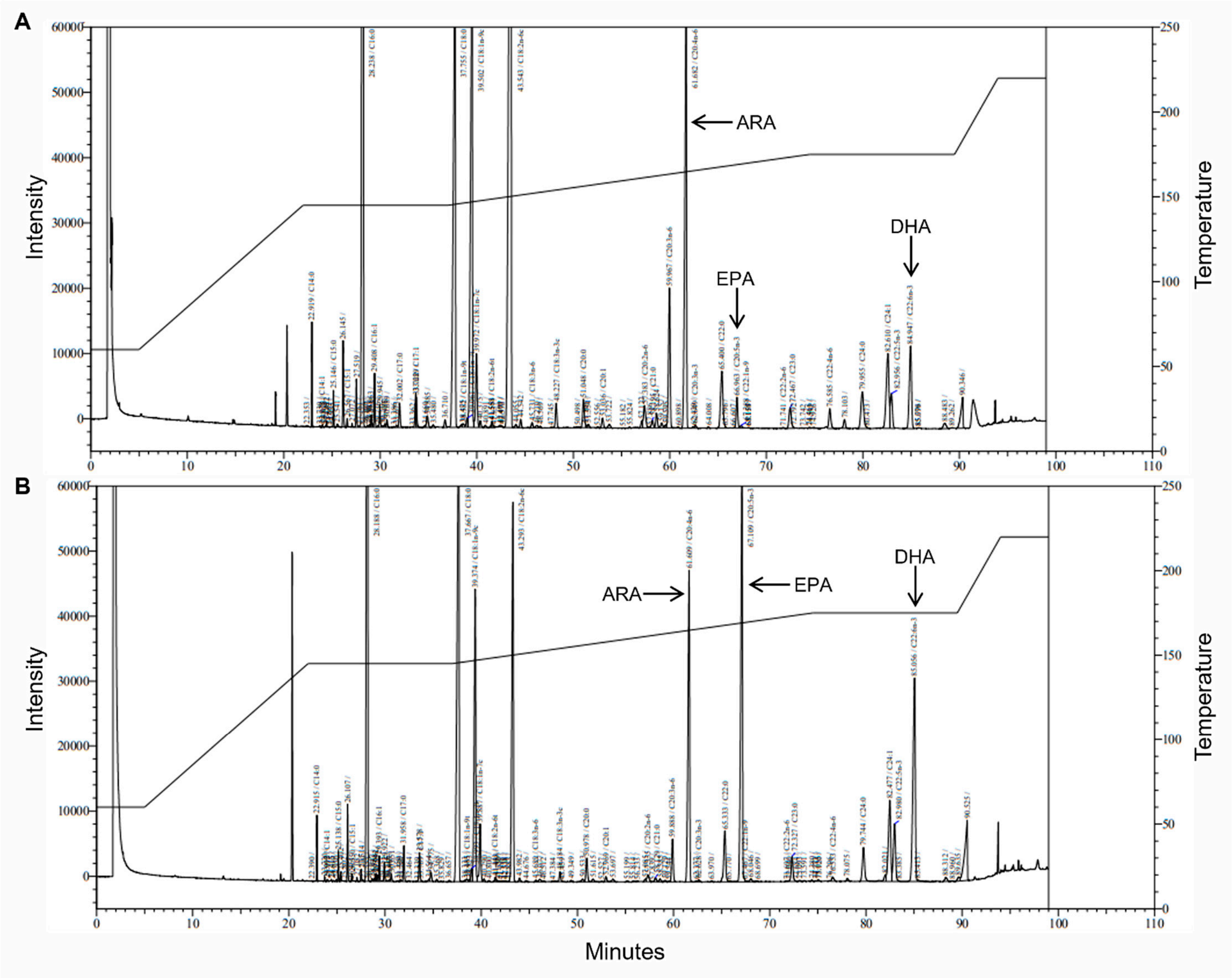

Figure S1. Typical chromatograms of plasma phospholipid fatty acid methyl esters for a patient that complied with the control supplementation (A) and for one that complied with the fish oil supplementation (B). Fatty acids were identified using the retention times of standards and GCsolution software (Shimadzu, France) was used to compute their area% (equation1), i.e., the area under the curve of the chromatographic peak expressed relative to the sum of the areas of all the chromatographic peaks during a predetermined time window. The fatty acids identified accounted for more than 95% of the area of all the chromatographic peaks. For each sample, the integration window and identification of each fatty acid were verified and adjusted by the same person (C.G.). EPA, eicosapentaenoic acid (20:5n-3); DHA, docosahexaenoic acid (22:6n-3); ARA, arachidonic acid (20:4n-6).

Figure S2

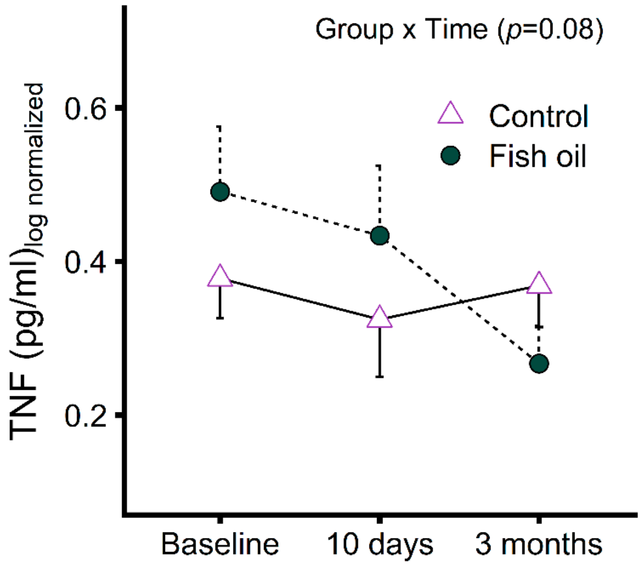

Figure S2. Plasma tumor necrosis factor alpha (TNF) in advanced breast cancer patients who complied with fish oil supplementation (*n* = 14) or with a control supplementation (*n* = 17) throughout the trial.

Figure S3

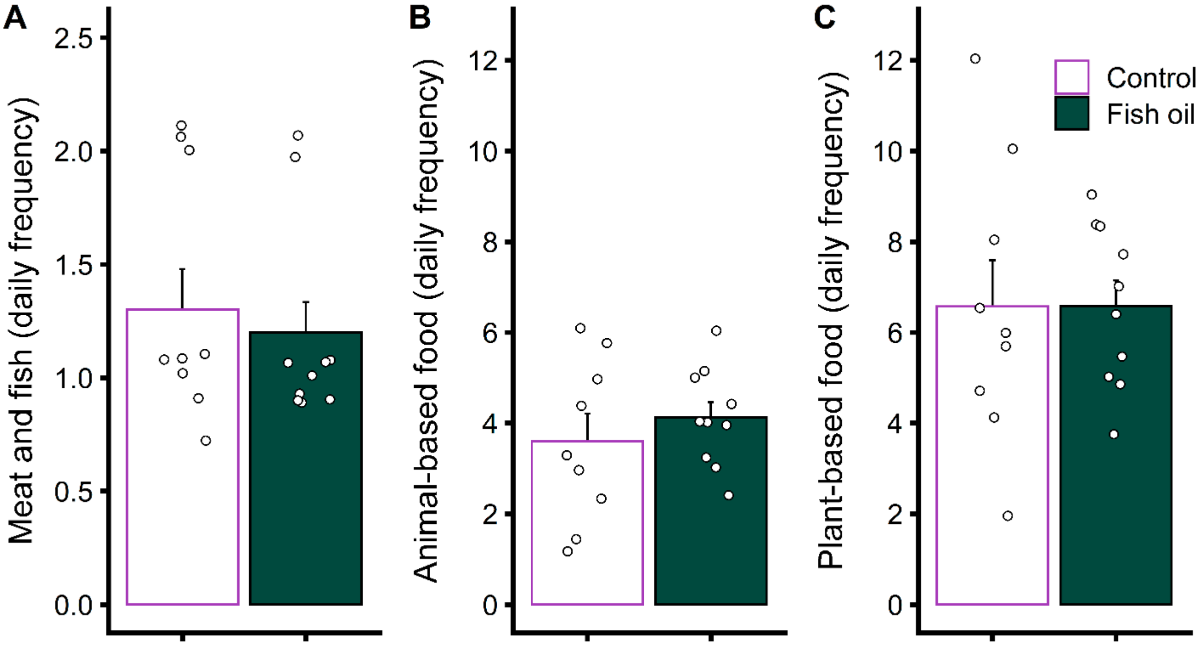

Figure S3. Reported frequencies of meat and fish intake (A) and indices of animal-based (B) and plant-based (C) food intakes in the subsample of advanced breast cancer patients used for isotopic analyses (fish oil supplementation: $n = 10$, control supplementation: $n = 9$). No participant reported not consuming meat or seemed to avoid animal proteins. Because of the small sample size and the qualitative nature of the approach, these analyses should be interpreted with caution. More work on this topic is required. Plasma $\delta^{15}N$ was not associated with intake frequencies. We found an association between baseline plasma $\delta^{13}C$ and animal-based food intake index ($R = 0.64$, $p < 0.01$, Figure S4), corroborating literature data on the topic (Votruba et al., 2019, Yun et al., 2018). Meat and fish intake was the reported daily frequency of meat, ham, fish, or egg intake assessed through one question in the food frequency questionnaire. Animal-based food consumption index was calculated as the sum of meat and fish intake and of the reported daily frequencies of dairy product intake (e.g., milk, yoghurt, cheese, etc.) assessed through 3 questions in the food frequency questionnaire. Plant-based food consumption index was calculated as the sum of the reported daily frequencies of plant-based food intake (e.g., bread, pasta, legumes, vegetables, fruits, rice, etc.) assessed through 5 questions in the food frequency questionnaire.

Figure S4

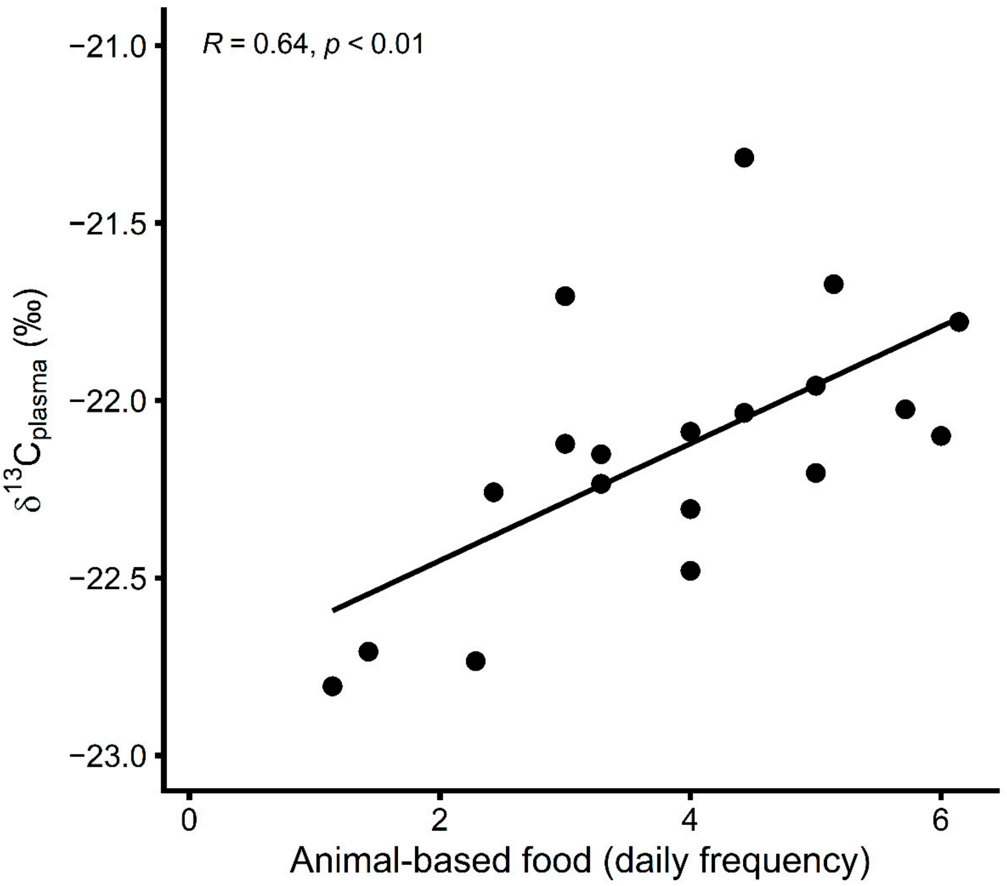

Figure S4. Association between animal-based food consumption index and baseline plasma $\delta^{13}C$ in the subsample of advanced breast cancer patients used for isotopic analyses (*n* = 19). This association, corroborating literature data on the topic (Votruba et al., 2019, Yun et al., 2018), seems to be mostly driven by dairy products. Animal-based food consumption index was calculated as the sum of the reported frequencies of meat, ham, fish, or egg intake assessed through one question in the food frequency questionnaire and of dairy product intake (e.g., milk, yoghurt, cheese, etc.) assessed through 3 questions in the food frequency questionnaire.

Figure S5

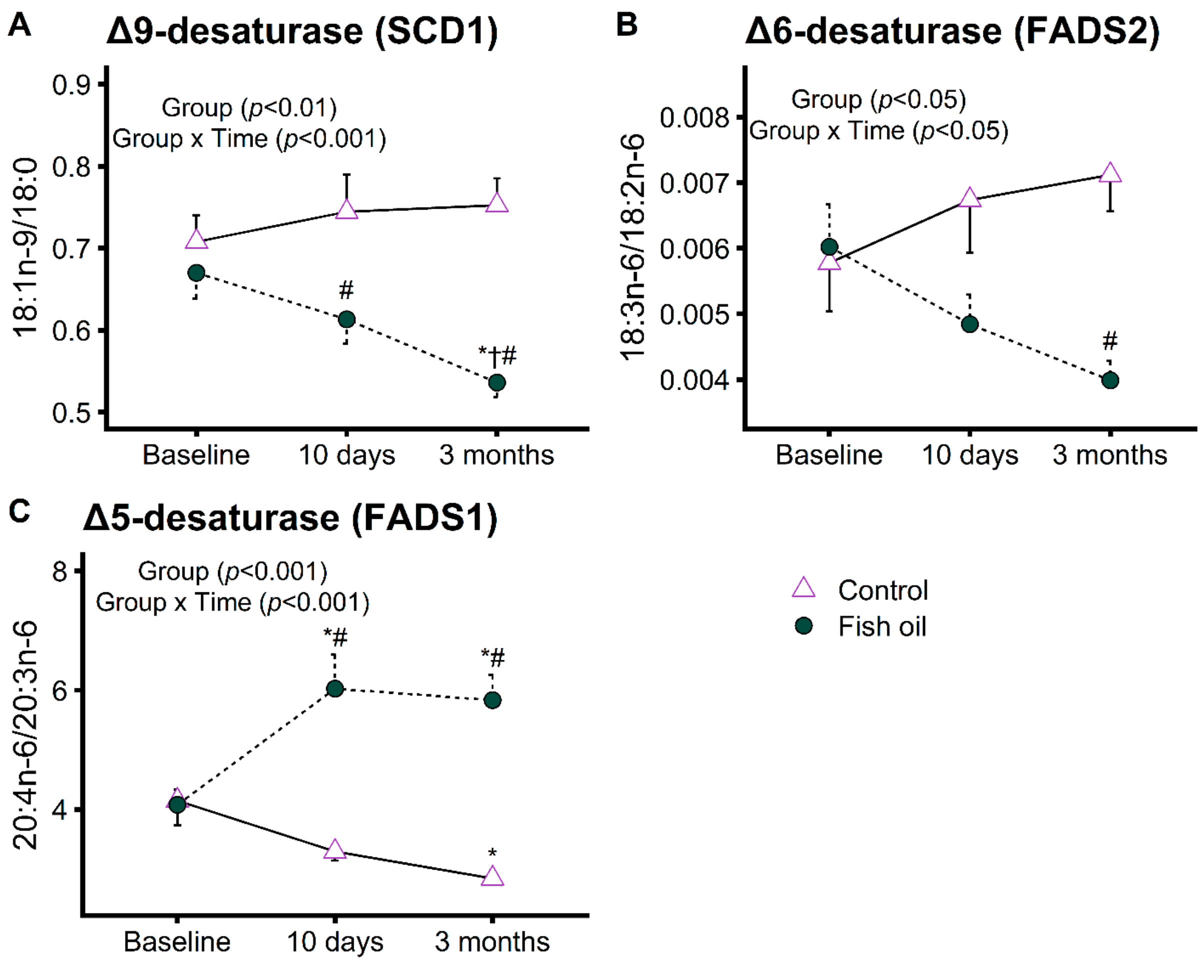

Figure S5. Indices of enzymatic activity in advanced breast cancer patients who complied with fish oil supplementation (n = 14) or with a control supplementation (n = 17) throughout the trial. Plasma ratios between precursors and products were used to estimate the activity of (A), Stearoyl-CoA desaturase 1 (SCD1, Δ9-desaturase)(18:1n-9/18:0), (B) fatty acid desaturase 2 (FADS2, Δ6-desaturase)(18:3n-6/18:2n-6), (C) fatty acid desaturase 1 (FADS1, Δ5-desaturase)(20:4n-6/20:3n-6). *Significant difference with baseline values ($p < 0.05$). †Significant difference with values at 10 days ($p < 0.05$). #Significant difference with control ($p < 0.05$).
